# Does the Presence of Cognitive Impairment Exacerbate the Risk of Falls in People with Peripheral Neuropathy? An Application of Body-Worn Inertial Sensors to Measure Gait Variability

**DOI:** 10.3390/s20051328

**Published:** 2020-02-29

**Authors:** Gu Eon Kang, Jacqueline Yang, Bijan Najafi

**Affiliations:** Interdisciplinary Consortium on Advanced Motion Performance (iCAMP), Michael E. DeBakey Department of Surgery, Baylor College of Medicine, Houston, TX 77030, USA; gueon.kang@bcm.edu (G.E.K.); jacqueline.yang@bcm.edu (J.Y.)

**Keywords:** Cognitive impairment, diabetic peripheral neuropathy, chemotherapy-induced peripheral neuropathy, single-task walking, dual-task walking, gait variability, body-worn sensors

## Abstract

People with peripheral neuropathy (PN) are at risk of falling. Many people with PN have comorbid cognitive impairment, an independent risk factor of falls, which may further increase the risk of falling in people with PN. However, the negative synergic effect of those factors is yet to be reported. We investigated whether the presence of cognitive impairment exacerbates the risk of falls in people with PN by measuring gait variability during single-task walking and dual-task walking. Forty-four adults with PN were recruited. Based on the Montreal Cognitive Assessment (MoCA) scores, 19 and 25 subjects were cognitively impaired and intact, respectively. We measured coefficients of variation of gait speed, stride length, and stride time using validated body-worn sensors. During single-task walking, no between-group differences were observed (all *p* > 0.05). During dual-task walking, between-group differences were significant for gait variability for gait speed and stride length (51.4% and 71.1%, respectively; *p* = 0.014 and 0.011, respectively). MoCA scores were significantly correlated with gait variability for gait speed (*r* = 0.319, *p* = 0.035) and stride length (*r* = 0.367, *p* = 0.014) during dual-task walking. Our findings suggest that the presence of cognitive impairment exacerbates the risk of falls in people with PN.

## 1. Introduction

Peripheral neuropathy (PN), causing numbness in the foot, affects more than 20 million people in the United States [1]. People with PN are prone to falls [2,3], resulting in reduced quality of life and increased mortality [4,5]. Statistics show that people with PN have up to 23 times higher risk of falls compared to those without [6]. Although the numbness in the foot is considered a major risk factor for falls [7,8,9,10], a recent review suggests that the numbness may not solely explain the high risk of falls [11]. For example, one study reported that people with type 2 diabetes and foot numbness had similar postural sway, an indicator of the risk of fall, when compared to people with type 2 diabetes but no foot numbness [12]. Thus, in order to fully understand the mechanism of the frequent falls in people with PN, it is necessary to identify other specific factors than foot numbness that contribute to falls.

Cognitive impairment is prevalent in people with PN [13,14,15,16] and a well-established risk factor for falls in the general population [17,18]. For example, cognitively impaired older adults have two times higher risk of falls compared to cognitively intact older adults [19]. Despite the association between cognitive impairment and increased risk of falls in other populations, its effect on the risk of falls in people with PN remains elusive. Based on findings in other populations [17,18], it is reasonable to hypothesize that the presence of cognitive impairment in addition to PN may exacerbate the risk of falls. 

Investigating walking under cognitively demanding conditions, i.e., dual-task walking, may be a proper candidate test to identify the effect of cognitive impairment on the risk of falls in people with PN. As walking is not just an automatic physical task but an attentional-demanding task of continuous postural control, an additional cognitive task interferes with the attention of postural control during walking [20,21]. Previous studies have reported the impact of cognitive status on dual-task walking in the general population or patient populations other than those with PN [22,23,24,25,26]. For example, the increase in gait variability from single-task walking, in which no other task but walking was demanded, to dual-task walking was significantly greater for people with cognitive impairment (without neurological or orthopedic disorder) compared to people without cognitive impairment [25]. In stroke survivors, gait variability increased with cognitive impairment [26]. Although these previous studies did not directly report dual-task walking performance in people with PN, they provided implications that gait variability may worsen with cognitive impairment in PN.

In terms of equipment to assess gait variability during single-task walking and dual-task walking, previous studies utilized an optoelectronic motion capture system [27,28] or instrumented walkway [29,30]. Although their validities and accuracies are well established, their high cost and restricted capture volume (e.g., captures between 5 and 10 m) limit their applications, as continuously discussed in previous reports [31,32]. Furthermore, they are not appropriate outside a gait laboratory such as in busy clinical settings and home settings, which further limits translating the outcomes for clinical use. In order to address these limitations from so-called “conventional” methods, body-worn inertial sensors that are low-cost, not restricted by capture volume, and have a potential for clinical translation (i.e., eligibility for translating outcomes) are more appropriate to study gait variability associated with cognitive impairment. 

The overall objectives of this study were to investigate whether or not the presence of cognitive impairment increases the risk of falls in people with PN. In order to assess risk of falls, we measured gait variability during single-task walking and dual-task walking using body-worn inertial sensors. Based on previous findings about the effect of cognitive impairment on gait variability in other populations [25], we hypothesized that people with PN and cognitive impairment would have greater gait variability (i.e., more unsteady gait) during single-task walking and dual-task walking compared to people with PN without cognitive impairment. We also hypothesized that people with PN and cognitive impairment, when compared to people with PN without cognitive impairment, would have a greater increase in gait variability from single-task walking to dual-task walking.

## 2. Materials and Methods

### 2.1. Participants

This study is an additional analysis of two datasets in which walking performance in people with foot numbness because of PN due to either diabetes mellitus or chemotherapy was assessed. Originally, motion data from 31 people with PN due to diabetes mellitus and 35 people with PN due to chemotherapy were available. Among them, we included participants whose data for cognitive status, single-task walking, and dual-task walking were wholly available. In addition, in order to rule out any confounding effects from neurological disorders outside of PN, we excluded participants with a history of additional neurological disorders such as Parkinson’s disease, stroke, and dementia. As a result, we included data from 44 participants. Then, the 44 people with PN were classified into two groups: PN without cognitive impairment (*N* = 25) and PN with cognitive impairment (*N* = 19).

All participants were recruited from outpatient podiatry or oncology clinics located of the Greater Houston Area in Texas and had foot numbness that was caused by type II diabetes mellitus or chemotherapy. PN diagnoses were confirmed by the participants’ physicians. All participants were able to walk independently. The experimental protocols were conducted in the Gait Laboratory of the Interdisciplinary Consortium on Advance Motion Performance at the Baylor College of Medicine in accordance with the regulations and guidelines of the Declaration of Helsinki, and they were approved by the Institutional Review Board at the Baylor College of Medicine. We obtained written informed consent from all participants prior to their participation.

### 2.2. Physiological, Psycho-Cognitive, and Functional Assessments

We assessed the severity of PN by measuring vibration perception threshold (VPT) in the plantar surface of the foot with a standard Biothesiometer (Bio-Medical Instrument, Newbury, Ohio) as performed previously [33,34,35,36,37]. For each participant, we measured VPT for the first and fifth metatarsal heads and heel in each foot with the probe of the Biothesiometer. We gradually increased the vibration from 0 V until participants began feeling the vibration. We then continued to increase the vibration slightly and began decreasing the vibration until participants could no longer feel any vibration. When the difference between two vibrations was less than 2 V, the results were accepted. The greater value was considered the VPT value for the site of evaluation. VPT was the maximum value among the six VPT values, and it was considered as the severity of PN.

We measured cognitive status using the Montreal Cognitive Assessment (MoCA) [38]. MoCA assesses multiple cognitive domains of visuospatial and executive functions, memory, attention, and language with a score ranging between 0 and 30. In the current study, we used the cutoff score of 22 and below to identify those with cognitive impairment [39]. MoCA was used to identify dual-task related motor dysfunction previously [40,41]. Additionally, we assessed for depression in each participant using the Center for Epidemiological Studies Depression Scale (CES-D) [42]. CES-D is a 20-item self-administered questionnaire that evaluates symptoms associated with depression such as sleep, appetite loss, and loneliness. Scores for each item range from 0–3 (a total of 60), and someone with a score of 16 and over in the CES-D is considered at risk for depression. We also assessed fear of fall using the Falls Efficacy Scale International (FES-I) [43]. FES-I is a 16-item self-administered questionnaire. Scores for each item range from 1–4 (a total of 64), and someone with a score of 20 and over on the FES-I is considered at moderate to high fear of fall [44]. Participants also provided information about their history of fall in the past 12 months.

### 2.3. Walking Performance

We evaluated walking performance under single-task and dual-task conditions in a hallway at the Baylor College of Medicine McNair building located in Houston, Texas. Each participant began walking from an upright standing position at a comfortable pace for them. For the single-task walking condition, participants were instructed to walk as comfortably and normally as possible. For the dual-task walking condition, participants were instructed to count backward from a random number specified by clinical staff while walking. For both walking conditions, participants walked approximately 12 m (40 feet) in the hallway.

While participants were walking, we collected movement data using two commercially available inertial sensors (LegSysTM, BioSensics, Watertown, Massachusetts) worn on the distal end of the anterior surface of the shanks using flexible straps. Each inertial sensor was composed of an accelerometer, a gyroscope, and a magnetometer that collected the linear acceleration and angular velocity of the body segment on which the sensor was worn. The sampling frequency of each sensor was set at 100 Hz. The locations and algorithms of the inertial sensors for measuring spatiotemporal gait parameters (gait speed, stride length, stride time) and gait variability of the spatiotemporal gait parameters were validated in previous studies [32,45,46,47].

### 2.4. Data Analysis

The primary outcome was gait variability. For both walking conditions, we calculated the coefficient of variation (CV) of gait speed, stride length, and stride time. CV was calculated as the ratio of the standard deviation to the mean, expressed as a percentage. In addition, for both walking conditions, we calculated spatiotemporal gait parameters such as gait speed, stride length, and stride time.

### 2.5. Statistical Analysis

Between the two groups, we compared demographic and clinical characteristics using independent sample *t*-tests for body mass index, MoCA scores, and VPT, Mann–Whitney U tests for age, FES-I scores, CES-D scores, and number of falls, and chi-square tests for number of women, participants who fell in the last 12 months, participants at risk of depression, and participants with moderate to high fear of fall.

Between the two groups, we compared gait speed, stride length, stride time, and CV of gait speed, stride length, and stride time for each walking condition using analysis of covariance (ANCOVA), accounting for potential effects of age, body mass index, and sex on the outcomes. Bonferroni correction was applied for ANCOVA tests. In addition, between the two walking conditions, we compared the outcomes for each group using a linear mixed model with random effects of participants and fixed effects of age, body mass index, sex (i.e., covariates), and walking conditions (i.e., single-task and dual-task). Lastly, we investigated correlations between MoCA scores and the primary outcomes (i.e., CV of gait speed, stride length, and stride time) across all participants using Spearman’s correlation (*r_s_*). For all statistical analyses, a *p*-value less than 0.05 was considered statistically significant. We also calculated effect size using Cohen’s *d*, and denoted it as *d*. Effect size was classified as follows: *d* ≤ 0.19 = no noticeable effect; 0.20 ≤ *d* ≤ 0.49 = small effect; 0.50 ≤ *d* ≤ 0.79 = medium effect; *d* ≥ 0.80 = large effect [48]. We used SPSS® version 25 (IBM, Armonk, New York) for statistical analysis.

## 3. Results

### 3.1. Participant Characteristics

Table 1 shows participant demographic and clinical characteristics. As expected, MoCA scores were significantly higher in the cognitively intact group compared to the cognitively impaired group (*p* < 0.001). However, there was no significant difference between the two groups for age, body mass index, sex, VPT, CES-D, FES-I, history of falls, high risk of depression, or moderate to high fear of fall (all *p* > 0.05).

### 3.2. Walking Performance between the Cognitively Intact and Impaired Groups

Table 2 shows walking performance between the two groups for each walking condition. During single-task walking, we found no significant differences for any of the gait variables between the two groups (all *p* > 0.05; all *d* ≤ 0.50). In contrast, during dual-task walking, we found significant differences between the two groups for CV of gait speed and stride length (*p* = 0.014 and 0.011, respectively; *d* = 0.87 and 0.89, respectively). Stride length was marginally different between the two groups for dual-task walking (*p* = 0.060; *d* = 0.57). However, differences in gait speed, stride time, and CV of stride time during dual-task walking between the two groups did not reach statistical significance (all *p* > 0.05; all *d* ≤ 0.53).

### 3.3. Walking Performance between Normal and Dual-Task Conditions

**Figure 1:**
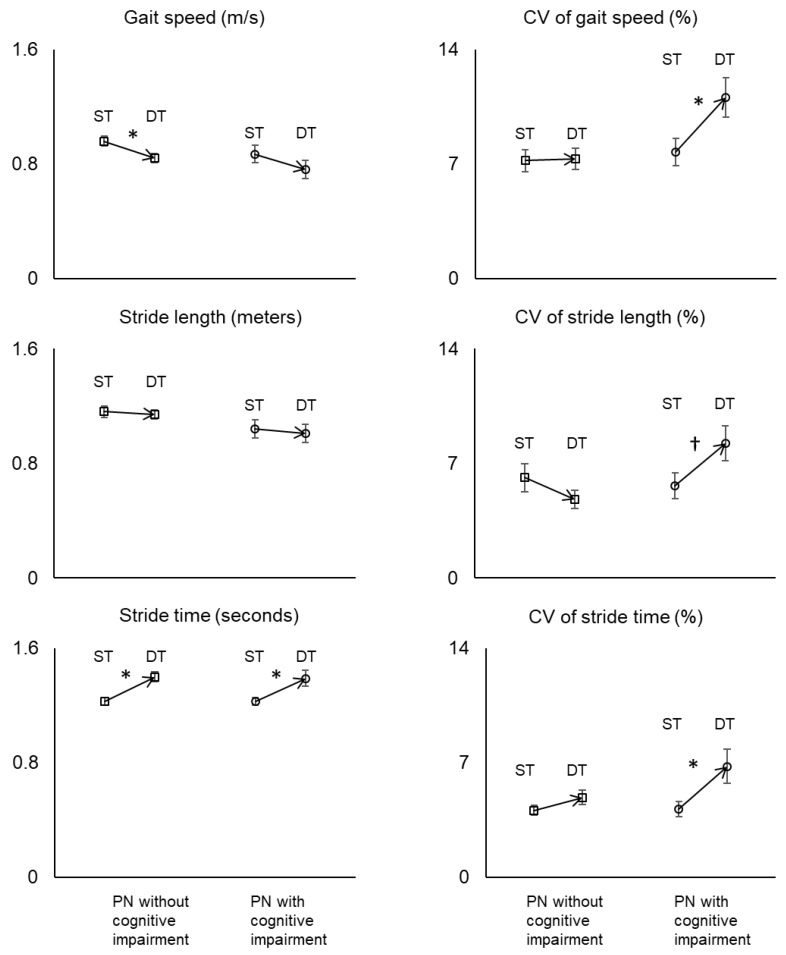
Changes in gait parameters and CV of the parameters from single-task walking (ST) to dual-task walking (DT) for the PN without cognitive impairment group and the PN with cognitive impairment group. Asterisks denote significant changes in DT from ST (all *p* < 0.05). The cross denotes a marginal change in DT from ST (*p* = 0.063). All significant changes were after accounting for the effects of age, body mass index, and sex.

Figure 1 shows changes in walking performance between the two walking conditions for each group. For the PN without cognitive impairment group, mean gait speed decreased by 12.5% and stride time increased by 13.8% for the dual-task walking condition compared to the single-task walking condition (*p* = 0.015 and <0.001, respectively; *d* = 0.70 and 1.12, respectively). However, stride length remained almost the same between the two walking conditions (*p* = 0.791; *d* = 0.11). For the PN without cognitive impairment group, gait variability assessed based on CV of gait speed, stride length, and stride time was not significantly different between the single-task and dual-task walking conditions (*p* = 0.922, 0.791, and 0.151, respectively; *d* = 0.03, 0.36, and 0.42, respectively).

For the PN with cognitive impairment group, mean stride time increased by 13.0% for the dual-task walking condition when compared to the single-task walking condition (*p* = 0.014; *d* = 0.84). Mean gait speed slightly increased, but the increase did not reach the statistical significance (*p* = 0.255; *d* = 0.40). Like the cognitively intact group, stride length remained almost the same between the two walking conditions (*p* = 0.684; *d* = 0.11). For the PN with cognitive impairment group, gait variability was significantly or marginally increased from the single-task walking condition to dual-task walking condition. CV of gait speed, stride length, and stride time increased by 43.2%, 46.4%, and 62.2%, respectively, for the dual-task walking condition when compared to the single-task walking condition (*p* = 0.031, 0.063, and 0.035, respectively; *d* = 0.74, 0.63, and 0.73, respectively).

### 3.4. Correlations between Cognitive Function and Stride-to-Stride Variability

**Figure 2:**
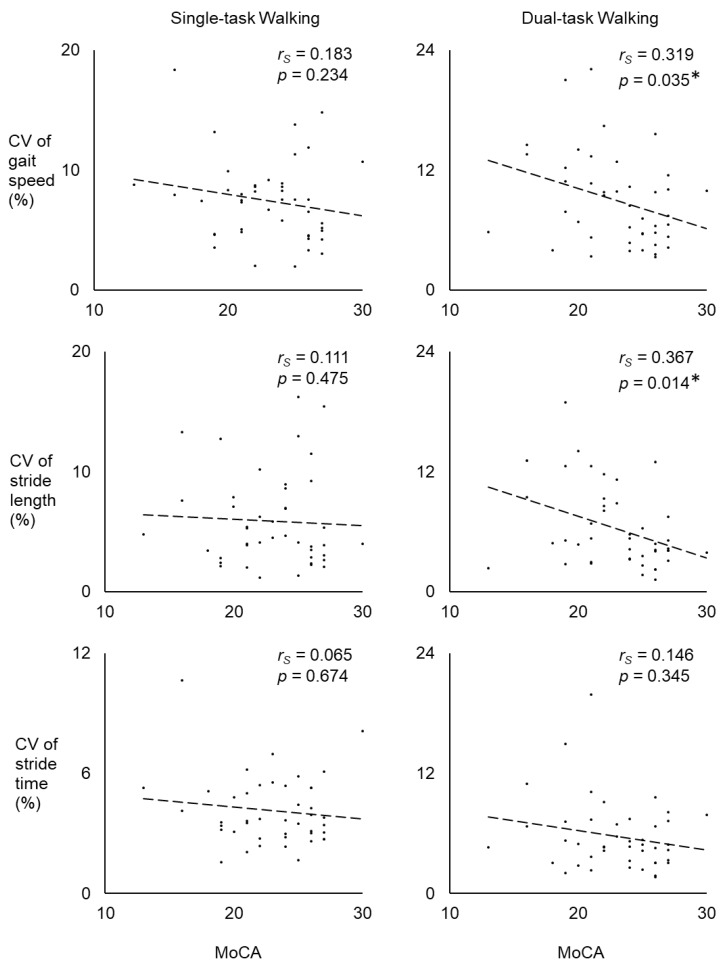
Spearman correlations (r_s_) between MoCA scores and CV in gait parameters across all participants during single-task walking and dual-task walking. Asterisks denote significant correlations between MoCA scores and gait variables.

Figure 2 shows correlations between MoCA scores and CV of gait speed, stride length, and stride time for each walking condition across all participants. There were no significant correlations between MoCA scores and gait variability during single-task walking (all *p* > 0.05; all *r_s_* < 0.200). For the dual-task walking condition, CVs of gait speed and stride length were significantly correlated with MoCA scores (*p* = 0.035 and 0.014, respectively; *r_s_* = 0.319 and 0.367, respectively). However, CV of stride time was not significantly correlated with MoCA score during dual-task walking (*p* = 0.345; *r_s_* = 0.146).

## 4. Discussion

In this study, we investigated whether the presence of cognitive impairment would increase gait variability in people with PN by examining CV of gait speed, stride length, and stride time during single-task and dual-task walking. We found that people with PN with cognitive impairment had more variability in gait speed and stride length (i.e., a more unsteady gait) during dual-task walking when compared to people with PN without cognitive impairment, but that, during single-task walking, there was no statistical difference between the two groups. Another primary finding was that, within the PN with cognitive impairment group, gait variability significantly increased from single-task walking to dual-task walking; however, within the PN without cognitive impairment group, gait variability did not significantly change from single-task walking to dual-task walking. Additionally, MoCA scores were significantly correlated with CV of gait speed and stride length during dual-task walking. This suggests that the presence of cognitive impairment exacerbates the risk of falls in people with PN and that cognitive impairment is an additional cause of falls to the somatosensory deficits.

As hypothesized, increases in CV of gait speed, stride length, and stride time from single-task walking to dual-task walking were greater for the group of foot numbness (i.e., PN) with cognitive impairment than for the group of foot numbness without cognitive impairment, and they tended to be significant only for the group of foot numbness and cognitive impairment. These results suggest that the effects of dual tasks while walking on gait variability were significant only for the cognitively impaired people with PN. One possible reason for these results is the impairment in the postural compensatory strategies. A previous study suggested that the foot numbness results in deteriorated postural compensatory strategies during a motor task in people with PN [49]. The deteriorated postural compensatory strategies would have resulted in heavy reliance on cognitive function during walking in order to avoid falls. However, when one has cognitive impairment in addition to foot numbness and an additional cognitively demanding task is given, this mechanism is disturbed, which is manifested as significant increases in gait variability. Our findings are in line with previous work that reported significant increases in stride time variability in people with mild cognitive impairment [25,50], as well as stride length variability in people with diabetes mellitus (with and without PN) with cognitive impairment [51]. Although both groups performed similarly during single-task walking, the dual-task condition seems to have played a significant role in interfering with the attention of balance control for the cognitively impaired group.

One implication of a more unsteady gait in cognitively impaired people with PN is risk of fall [52], a major cause of death and loss of independence [53]. Falls during walking often occur when an additional task such as talking is being performed in conjunction with walking, and the attention to walking is distracted [54]. Taken together, the significant increases in gait variability during the dual-task walking condition compared with the single-task walking condition in people with PN and cognitive impairment suggest higher risk of falls compared to those without cognitive impairment.

Results from this study emphasize the necessity of the systematic and continuous monitoring of somatosensory and cognitive declines, as these independently contribute to the increased risk of falls in people with PN. The monitoring would be possible through digital health technology such as smart shoes or smart socks. For example, gait variables examined in this study can be embedded in smart shoes or smart socks, a topic that has been discussed in a recent article [55]. This would allow the clinical care team to continuously monitor changes in the risk of fall during day-to-day activities, and it may be possible to inform the team any notable changes that are likely to increase the risk of falls.

One limitation in our study was small sample size. Due to the small sample size, it was not possible to investigate if a sub-component of the cognitive domains such as visuospatial and executive functions, attention, and memory is the main cause of increasing gait variability. Future studies with a larger sample size are recommended in order to confirm generalizability of our results. Another limitation is that we included people with PN due to two different causes, type II diabetes mellitus and chemotherapy, in this study. The two causes may have had different neurodegenerative effects on cognition that could have affected performance during the dual-task walking condition. It is recommended that clinicians and researchers investigate effects of cognitive impairment due to each of these causes on walking performance in future studies.

## 5. Conclusions

We demonstrated that the presence of cognitive impairment exacerbates the risk of falls in people with PN. We confirmed that cognitive impairment in people with PN increased gait variability only for the dual-task walking condition and not for the single-task walking condition. Since we used body-worn sensors in this study, we believe that outcomes from this study have the potential to be implemented in digital health technology. Based on our findings, we propose for clinicians to consider people with PN and cognitive impairment at a higher risk for fall as compared to those with PN but no cognitive impairment.

## Figures and Tables

**Table 1:** Participant characteristics for each group. PN—peripheral neuropathy; VPT—vibration perception threshold; MoCA—Montreal Cognitive Assessment; CES-D—Center for Epidemiological Studies Depression Scale; FES-I—Falls Efficacy Scale International.

Measures	PN without Cognitive Impairment (*N* = 25)	PN with Cognitive Impairment (*N* = 19)	*p*-Value
Age, years	66.5 ± 9.1	68.5 ± 9.1	0.324
Body mass index, kg/m^2^	31.3 ± 5.9	29.0 ± 6.2	0.228
Men:Women, *N*	15:10	8:11	0.888
VPT, volts	26.3 ± 12.7	27.2 ± 12.1	0.813
MoCA	25.6 ± 1.6	19.6 ± 2.4	<0.001 *
CES-D	6.6 ± 8.2	9.1 ± 6.7	0.125
Risk of depression, *N*	2	4	0.184
FES-I	29.3 ± 14.7	35.2 ± 14.0	0.073
Moderate to high fear of fall, *N*	17	17	0.092
People who fell in the past year, *N*	8	7	0.737
Number of falls in the past year, *N*	0.9 ± 2.1	0.6 ± 1.1	0.866

*Note*: Variables are expressed as means ± standard deviation. Moderate to high fear of fall represents people that had FES-I ≥ 20. Risk of depression represents people that had CES-D ≥ 16. The asterisk denotes a significant between-group difference (*p* < 0.05).

**Table 2:** Gait parameters and variability during single-task walking and dual-task walking for each group. CV—coefficient of variation.

Measures	PN without Cognitive Impairment	PN with Cognitive Impairment	*p*-Value	Cohen’s *d*
Single-task walking				
Gait speed, m/s	0.96 ± 0.18	0.87 ± 0.27	0.236	0.39 ^S^
Stride length, meters	1.16 ± 0.19	1.04 ± 0.28	0.160	0.50 ^M^
Stride Time, seconds	1.23 ± 0.13	1.23 ± 0.12	0.855	0.00 ^N^
CV of gait speed, %	7.21 ± 3.34	7.73 ± 3.63	0.804	0.15 ^N^
CV of stride length, %	6.12 ± 4.29	5.62 ± 3.48	0.615	0.13 ^N^
CV of stride time, %	4.10 ± 1.59	4.18 ±1.99	0.905	0.04 ^N^
Dual-task walking				
Gait speed, m/s	0.84 ± 0.16	0.76 ± 0.28	0.300	0.35 ^S^
Stride length, m	1.14 ± 0.16	1.01 ± 0.28	0.060 †	0.57 ^M^
Stride Time, s	1.40 ± 0.17	1.39 ± 0.24	0.937	0.05 ^N^
CV of gait speed, %	7.31 ± 3.20	11.07 ± 5.22	0.014 *	0.87 ^L^
CV of stride length, %	4.81 ± 2.80	8.23 ± 4.66	0.011 *	0.89 ^L^
CV of stride time, %	4.89 ± 2.14	6.78 ± 4.60	0.119	0.53 ^M^

*Note*: Variables are expressed as means ± standard deviation. Asterisks denote significant between-group differences (*p* < 0.05). The cross denotes a marginal between-group difference. All significant differences were after accounting for the effects of age, body mass index, and sex. Superscript letters denote the following: N = no noticeable effect; S = small effect; M = medium effect; L = large effect.
